# Supplementary material for: Bayesian refinement of protein structures and ensembles against SAXS data using molecular dynamics
Source: PLoS Comput Biol. 2017 Oct 18;13(10):e1005800. doi: 10.1371/journal.pcbi.1005800 (PMC5662244; doi:10.1371/journal.pcbi.1005800)
Supplement: S1 Archive — (BZ2) [file pcbi.1005800.s011.tar.bz2 › gromacs_4.62_bayesian_saxs_refi/share/html/online/g_confrms.html]

g\_confrms


| g\_confrms Main Table of Contents |

**VERSION 4.6  
Sat 19 Jan 2013**

---

### Description

g\_confrms computes the root mean square deviation (RMSD) of two
structures after least-squares fitting the second structure on the first one.
The two structures do NOT need to have the same number of atoms,
only the two index groups used for the fit need to be identical.
With -name only matching atom names from the selected groups
will be used for the fit and RMSD calculation. This can be useful
when comparing mutants of a protein.

The superimposed structures are written to file. In a .pdb file
the two structures will be written as separate models
(use rasmol -nmrpdb). Also in a .pdb file, B-factors
calculated from the atomic MSD values can be written with -bfac.

### Files

| option | filename | type | description |
| --- | --- | --- | --- |
| **-f1** | conf1.gro | Input | Structure+mass(db): tpr tpb tpa gro g96 pdb |
| **-f2** | conf2.gro | Input | Structure file: gro g96 pdb tpr etc. |
| **-o** | fit.pdb | Output | Structure file: gro g96 pdb etc. |
| **-n1** | fit1.ndx | Input, Opt. | Index file |
| **-n2** | fit2.ndx | Input, Opt. | Index file |
| **-no** | match.ndx | Output, Opt. | Index file |

### Other options

| option | type | default | description |
| --- | --- | --- | --- |
| **-[no]h** | bool | no | Print help info and quit || **-[no]version** | bool | no | Print version info and quit || **-nice** | int | 19 | Set the nicelevel || **-[no]w** | bool | no | View output .xvg, .xpm, .eps and .pdb files || **-[no]one** | bool | no | Only write the fitted structure to file || **-[no]mw** | bool | yes | Mass-weighted fitting and RMSD || **-[no]pbc** | bool | no | Try to make molecules whole again || **-[no]fit** | bool | yes | Do least squares superposition of the target structure to the reference || **-[no]name** | bool | no | Only compare matching atom names || **-[no]label** | bool | no | Added chain labels A for first and B for second structure || **-[no]bfac** | bool | no | Output B-factors from atomic MSD values |

---

http://www.gromacs.org  
gromacs@gromacs.org
